# Caste-Specific Differences in Hindgut Microbial Communities of Honey Bees (*Apis mellifera*)

**DOI:** 10.1371/journal.pone.0123911

**Published:** 2015-04-15

**Authors:** Karen M. Kapheim, Vikyath D. Rao, Carl J. Yeoman, Brenda A. Wilson, Bryan A. White, Nigel Goldenfeld, Gene E. Robinson

**Affiliations:** 1 Institute for Genomic Biology, 1206 W. Gregory Dr., University of Illinois at Urbana-Champaign, Urbana, IL 61801 United States of America; 2 Department of Entomology, 505 S. Goodwin Ave., University of Illinois at Urbana-Champaign, Urbana, IL 61801 United States of America; 3 Department of Biology, 5305 Old Main Hill, Utah State University, Logan, UT 84341 United States of America; 4 Department of Physics, 1110 W. Green St., University of Illinois at Urbana-Champaign, Urbana, IL 61801 United States of America; 5 Department of Animal and Range Sciences, Montana State University, P.O. Box 172900, Bozeman, MT 59717, United States of America; 6 Department of Animal Sciences, 1207 W. Gregory Dr., University of Illinois at Urbana-Champaign, Urbana, IL 61801 United States of America; 7 Department of Microbiology, 601 S. Goodwin Ave., University of Illinois at Urbana-Champaign, Urbana, IL 61801 United States of America; Wageningen University, NETHERLANDS

## Abstract

Host-symbiont dynamics are known to influence host phenotype, but their role in social behavior has yet to be investigated. Variation in life history across honey bee (*Apis mellifera*) castes may influence community composition of gut symbionts, which may in turn influence caste phenotypes. We investigated the relationship between host-symbiont dynamics and social behavior by characterizing the hindgut microbiome among distinct honey bee castes: queens, males and two types of workers, nurses and foragers. Despite a shared hive environment and mouth-to-mouth food transfer among nestmates, we detected separation among gut microbiomes of queens, workers, and males. Gut microbiomes of nurses and foragers were similar to previously characterized honey bee worker microbiomes and to each other, despite differences in diet, activity, and exposure to the external environment. Queen microbiomes were enriched for bacteria that may enhance metabolic conversion of energy from food to egg production. We propose that the two types of workers, which have the highest diversity of operational taxonomic units (OTUs) of bacteria, are central to the maintenance of the colony microbiome. Foragers may introduce new strains of bacteria to the colony from the environment and transfer them to nurses, who filter and distribute them to the rest of the colony. Our results support the idea that host-symbiont dynamics influence microbiome composition and, reciprocally, host social behavior.

## Introduction

Host-symbiont dynamics are emerging as a rich source of phenotypic diversity among individuals [1,2]. For example, variation in the community composition of gut bacteria is known to influence host nutrient acquisition [3,4], immune function [5,6], and mate preference [7]. Likewise, communities of symbiotic gut bacteria are reflective of variation in the phenotype of their hosts, and are influenced by host diet, immune activation, and their associated physiology [8,9]. Phenotypic diversity among individuals may thus be the result of a reciprocal interchange between host and symbiont, but we are only beginning to understand the dynamics of this relationship.

Social insect species provide excellent opportunities to explore the relationship between community composition of symbiotic bacteria and host phenotypic variation. In many species of ants, bees, and wasps, individuals within the same colony exhibit extreme differences in behavior, accompanied by variation in diet, nutritional status, physiology, and lifespan [10]. Honey bee (*Apis mellifera*) queens, for example, mate early in life and then remain in the hive specializing in egg production. Relative to the other female caste, the worker, the queen is larger with more developed reproductive organs and lives an order of magnitude longer. Workers specialize in brood care early in life as nurses, and transition toward the end of their 4–7 week adult life to working outside the hive as foragers, but do not mate and rarely reproduce. Males spend most of their early life resting or begging for food from nurses, and their behavior and physiology is centered on mating.

Some of the phenotypic differences among queens, workers, and males stem from variation in diet during development or as adults. Differential access to nutrients influences caste determination during the larval stage, with future queen larvae receiving more royal jelly than future workers [11]. As adults, queens receive diets high in protein, while the quality of worker diets varies over their lifespan [11,12]. Nurses eat a pollen-rich diet and feed nestmates inside the hive [13]. Foragers do not eat pollen, and though they receive some protein and lipids from nurses, they have low levels of nutrient stores and gut proteolytic enzymes [13,14]. Pollen consumption, proteolytic activity, and pollen digestion are all lower in males than workers [15]. These links between social behavior, diet, and gut physiology suggest there may also be a link between social behavior and the gut microbiota. However, differences in host-symbiont dynamics among the different castes in eusocial insect colonies have not yet been investigated.

A link between microbial symbionts and sociality in bees has been previously hypothesized [16–18]. Within social species, close physical interactions create the potential for colony-wide transmission of gut microbiota, suggesting there should be little microbiome variation among individual colony members [6,17,19–22]. Remarkably similar gut microbiomes have been observed for workers from different colonies, continents, and races of honey bees [17,23–28]. Gut microbiomes are also highly similar between 9- and 30-day-old workers, which likely represent sub-classes of workers (e.g. nurses and foragers, respectively) [20]. However, microbiome variation associated with workers engaged in nursing and foraging behavior has not been explicitly investigated. Furthermore, the microbiome of queens and males has not been characterized.

We explored the link between host social behavior and the microbiome by investigating bacterial community variation within honey bee colonies. We hypothesized that gut microbiomes within a honey bee colony vary with social role. Phenotypic variation among social insect castes could arise from variation in the microbiome if caste-specific traits are related to metabolic properties of gut microbes. Reciprocally, caste-related differences in environmental exposure to bacteria, immunity, access to resources, and intestinal environments may influence the community composition of gut bacteria. We explored these possibilities by comparing the microbiome composition among castes within and across honey bee colonies.

## Methods and Materials

### Sample collection

We collected queens, males, nurses, and foragers from honey bee (*A*. *mellifera*) colonies at the University of Illinois at Urbana-Champaign. No special permissions were required to collect from these colonies because they are owned by the university and *A*. *mellifera* is not an endangered or protected species. A single queen, six males, six foragers, and six nurses were collected from each of three typical colonies headed by naturally mated queens in September 2011. We collected seven additional foragers from a pollen feeder in an indoor flight chamber that housed two colonies in February 2011. We pooled hindguts of four individuals from this collection into one sample and analyzed three individuals separately. We also included samples of seven-day-old virgin queens that were collected in summer 2003 after being reared in a “queen bank” (a colony that is used to store queens). These non-reproductive queens were held in cages within a colony so they could be fed by nurses, but were not yet functioning as queens. We pooled hindguts of four individuals from this collection into one sample and analyzed four individuals separately. Individual bees were collected live and stored at -80°C. We removed the hindgut and its contents from each bee using sterile dissection, and stored this in PowerSoil-htp Bead Solution (Mo Bio, Carlsbad, CA) at -20°C.

### DNA extraction and sequencing of hindgut contents

We extracted DNA from individual hindguts using a PowerSoil DNA isolation kit (Mo Bio, Carlsbad, CA), following the manufacturer’s instructions with an additional bead-beating step (120 s at 6.5 Meters/s). The hypervariable V1–V3 region of the 16S rRNA gene was amplified by PCR using tagged 27f and 534r primers designed with some degenerate nucleotides [29]. This region of the 16S rRNA gene has been demonstrated to most accurately reflect the full length 16S rRNA gene [30], and is highly amenable to taxonomic reconstruction [31,32]. Amplicons were sequenced using MiSeq technology (2 × 300 nt reads). We performed a titration run with 65 samples pooled according to their DNA concentrations. We then re-normalized the 60 samples that yielded >1,100 high-quality pairs of reads for a full bulk run. The five samples that yielded fewer than 1,100 paired-end reads were failed PCR amplifications (as indicated by a lack of a band on the gel). These samples yielded few (24–325 paired-end) reads, and therefore served as negative controls: samples that failed to produce a band on a gel also failed to generate a robust microbiome profile. These five samples were excluded from further analysis.

### Bioinformatics pipeline

The total number of sequence reads obtained was 23,229,704 (11,614,852 pairs).

The reads were clustered into OTUs by implementing the steps used in the Tornado 2 metagenomics pipeline [33]. Since the read-pairs were overlapping, they were first merged using USEARCH v7.0 [34]. Of the 11,614,852 raw read-pairs, 9,242,697 (79.6%) were successfully merged. The OTU centers (consensus sequences) were first identified by dereplicating the merged reads and clustering with USEARCH at the default radius of 3.0 (corresponding to 97% sequence identity). This resulted in 112 OTU centers; these were then taxonomically classified using mothur [35] (with classify.seqs, using the Wang method and the Greengenes database). Those OTU centers that were unclassified at the species level were discarded, leaving a total of 83 OTUs. Finally, we used USEARCH (usearch_global command with the requirement of 97% sequence identity) to try to assign all the merged reads to one of the OTU centers. In this way, 6,252,345 merged reads were successfully assigned to one of the 83 OTUs.

### Classification of OTUs

We classified each sequence at the family level, and used blast [36] to identify those OTUs that had been previously found in bees (blastn, e-value <0.0001). We identified four OTUs as belonging to eukaryotes, and removed these from further analyses. OTUs classified as bacteria previously found in bees are denoted by the phylotype names first used by Babendreier *et al*. [37]. All OTUs belonging to each bee phylotype were from the same families: “alpha-1”—Bartonellaceae, “alpha-2.1”—Acetobacteraceae, “alpha-2.2”—Acetobacteraceae, “bifido”—Bifidobacteriaceae, “firm-4”—Lactobacillaceae, “firm-5”—Lactobacillaceae, “gamma-1”—Orbaceae, “gamma-2”—Orbaceae, “beta”—Neisseriaceae. Descriptions of some of these have since been published, in which case the genus name is presented following the phylotype name. The “beta” phylotype is *Snodgrassella alvi*. The “gamma” phylotypes and some unclassified sequences may include *Gilliamella apicola* (formerly “gamma-1”) [38], *Frischella perrera* (formerly “gamma-2”) [39], “gamma-3”, and “gamma-4” [24]. The “alpha-2.2” phylotype is *Parasaccharibacter apium* [40].

### Statistical analyses

To explore patterns of OTU diversity, a frequency table of OTUs in each sample was converted to a Bray-Curtis dissimilarity matrix using Primer 6 software with the PERMANOVA+ add-on package [41]. All downstream statistical analyses were based on the Bray-Curtis dissimilarity matrix, except where noted. We evaluated the unsupervised relationship between individual microbiomes with average-linkage clustering and non-metric multidimensional scaling (nMDS) in Primer 6. To describe the variation explained by the nMDS ordination, we calculated Pearson’s R^2^ for the correlation of sample distances in the reduced nMDS space with the original Bray-Curtis dissimilarities [42]. We calculated dispersion from the centroid with a dissimilarity-based multivariate extension of Levene’s test for homogeneity among individuals in each social caste with the PERMDISP function in Primer 6. We calculated the Shannon’s diversity index for each individual with the “diversity” function in vegan [43], and used these in an ANOVA in Stata (v. 9.2), followed by pairwise Bonferroni-corrected post-tests between castes in GraphPad. To partition the variance in the Bray-Curtis dissimilarity matrix, we used the semi-parametric PERMANOVA function in Primer 6 with caste as a fixed effect, colony as a random effect, and the interaction between colony and caste, with a type III partial sum of squares and Monte Carlo sampling permutated over residuals under a reduced model. This was followed by pairwise tests between castes with similar parameters, except that Monte Carlo permutations were unrestricted over raw data. This test is considered semi-parametric, because it is not based on an assumed distribution, and permutation techniques are thus used to obtain test statistics [41]. To test the effect of rare OTUs, we repeated the Bray-Curtis dissimilarity matrix and PERMANOVA analysis with log-transformed frequency data. This reduces the contribution of highly abundant OTUs relative to rare OTUs [41]. We evaluated the distinctness of each group with a supervised canonical analysis of principal coordinates (CAP) analysis in Primer 6 and leave-one-out cross validation. Canonical axes were calculated to maximize delimitation of caste groupings, and these axes were used to test the predictability of OTU frequency on caste membership. We also identified phylotypes for which abundance was significantly predicted by caste membership with negative binomial regression, using total read count as the exposure variable. We followed this with ANOVA in Stata (v 9.2) and pairwise Bonferroni-corrected post-tests between castes in GraphPad.

## Results

### Summary of sequences and classification

After filtering, we retained 6,251,931 of the total sequences, representing 79 OTUs. These sequences were distributed across 60 samples (4 queens, 16 males, 22 foragers, 18 nurses; 4 queen and 2 male samples were removed from analysis due to low PCR yields) (see S1 Fig for distribution of reads). The number of sequences was significantly different between castes (i.e., nurses, foragers, males, queens) (Kruskal-Wallis χ^2^ = 17.78, p = 0.0005, n = 60), with nurses having the most reads and queens having the least. The absolute amount of bacteria in bee hindguts was not addressed in this study, and will require further investigation. The variation in read count did not affect the number of OTUs detected in each group. Neither DNA concentration nor number of reads were significant predictors of OTU number in a negative binomial regression after removing the outlier sample with greater than 3.8-fold more reads than all other samples (LR χ^2^ = 3.02, n = 59, p = 0.22; S1 Fig). We detected an average of 26.4 ± 6.93 OTUs per individual. The number of OTUs detected was not significantly different between castes (Kruskal-Wallis χ^2^ = 4.85, p = 0.18, n = 60). The OTUs were classified into 44 taxonomic groups at the family level. Over 99% of the total sequences belonged to OTUs that were previously classified as honey bee phylotypes [37].

### Microbiomes differ between castes

**Fig 1:**
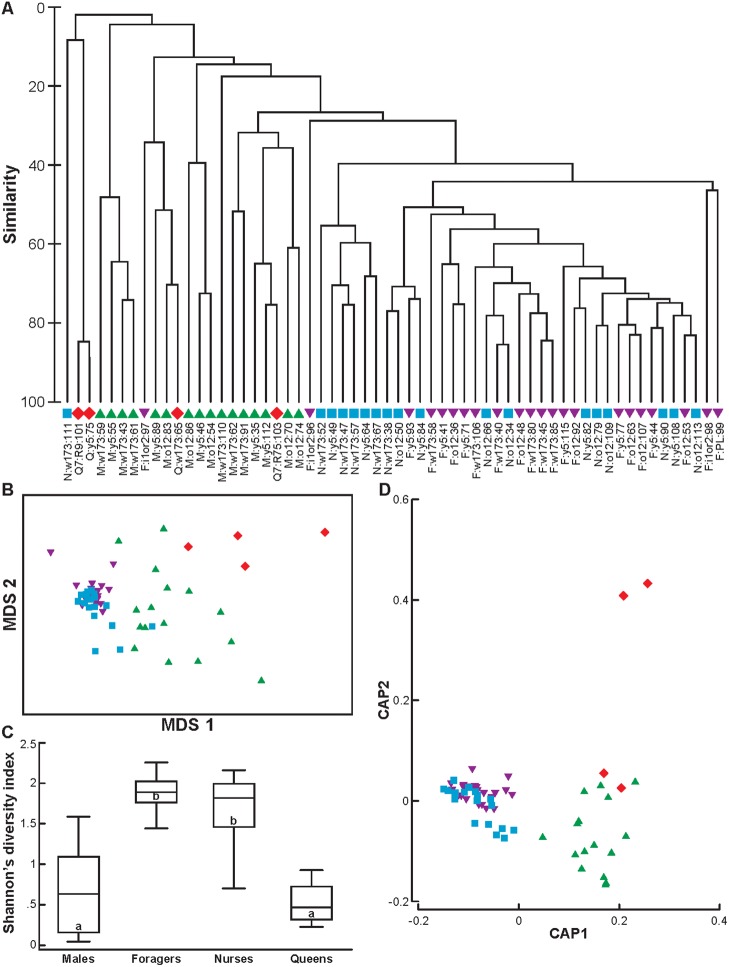
Individual microbiomes cluster by host caste in honey bees. Red diamonds represent queens; green triangles males; purple triangles worker foragers; light blue squares worker nurses. (A) Dendrogram from average-linkage clustering of OTU frequency. Leaf labels follow the format caste:colonyID:individualID. Q—queens from mature colonies, Q7—7d old queens, M—males, F—foragers, N—nurses; colony ‘i1or2’—indoor colony; PL—pooled sample from indoor colony. (B) Non-metric multi-dimensional scaling plot of OTU frequency after log-transformation, which reduces the influence of the most abundant OTUs. Stress value: 0.13 (C) Summary of Shannon’s diversity index based on OTU frequencies within social castes. Lines represent the median index value, boxes demarcate the interquartile range, whiskers are the adjacent values, and markers are the outer adjacent values. Statistical comparison was made with ANOVA; letters represent significant pairwise differences with p<0.05 in post-tests. (D) Canonical analysis of principal coordinates based on a Bray-Curtis dissimilarity matrix of OTU frequencies (non-transformed). Individual bees are plotted on canonical axes (CAP) 1 and 2, which are calculated to maximize delimitation among castes.

A separation of gut microbial communities among castes was revealed with average-linkage clustering (Fig 1A). All nurses and foragers from outdoor colonies, with the exception of one nurse, clustered together with greater than 40% similarity. The nurse that did not cluster with the other workers was an outlier with a small number of reads (S1 Fig). This sample clustered separately from all of the other samples. Although there was notable diversity among the male samples (i.e., they formed multiple clades with variable similarity), there were several clusters of males with high-similarity that did not include queens, nurses, or foragers. This pattern is supported by the non-metric multi-dimensional scaling (nMDS) plot, which shows overlap between nurses and foragers, and separation of these two types of workers from most males and queens (S2 Fig). The nMDS ordination yielded a two-dimensional solution with a final stress value of 0.13, which explained 91% of the variation in the raw data. Foragers from indoor and outdoor colonies and young and old queens did not form distinct groups, so we combined indoor and outdoor foragers and young and old queens for further downstream analyses.

Caste, colony, and the interaction of caste and colony were significant factors in a semi-parametric analysis of variance (S2 Table). Pairwise comparisons revealed that male hindgut bacterial communities were significantly different from workers (nurses and foragers), but not queens (Table 1). The hindgut bacterial communities of nurses and foragers were also significantly different from each other. We repeated the analysis of the full dataset after log-transformation, which reduced the contribution of highly abundant OTUs relative to rare OTUs. Differences between nurses and foragers were no longer statistically significant (Fig 1B, Table 2), indicating that differences between these groups were driven by differences in the proportion of highly abundant OTUs (e.g., *Lactobacillus* species Firm-4 and Firm-5). Log-transformation also revealed marginally significant differences between queens and workers (both nurses and foragers), suggesting that queens may have a unique rare microbiome.

**Table 1:** Results from pairwise comparisons of OTU variance between honey bee castes (PERMANOVA based on Bray-Curtis dissimilarity matrix of read counts).

	Males	Foragers	Nurses	Queens
**Males**		t = 2.69	t = 2.23	t = 1.08
		p = 0.0005	p = 0.0054	p = 0.4288
**Foragers**			t = 1.83	t = 1.45
			p = 0.0301	p = 0.1911
**Nurses**				t = 1.42
				p = 0.2172
**Queens**				

P-values are Monte-Carlo estimates from 9,999 unrestricted permutations of the raw data.

**Table 2:** Results from pairwise comparisons of OTU variance between honey bee castes with reduced influence of the most abundant OTUs (PERMANOVA based on Bray-Curtis dissimilarity matrix of log-transformed read counts).

	Males	Foragers	Nurses	Queens
**Males**		t = 3.16	t = 2.60	t = 1.48
		p = 0.0008	p = 0.0024	p = 0.1606
**Foragers**			t = 1.36	t = 2.34
			p = 0.1496	p = 0.0527
**Nurses**				t = 2.25
				p = 0.0512
**Queens**				

P-values are Monte-Carlo estimates from 9,999 unrestricted permutations of the raw data.

To investigate the role of differences in homogeneity within groups, we measured multivariate dispersion from individuals in each caste to their respective centroids. Results revealed significant differences in homogeneity overall (F = 22.83, p = 0.001, n = 60), and significant pairwise differences in homogeneity between males and both types of workers and between queens and both types of workers, but not between males and queens or nurses and foragers (Table 3). This indicates that differences in dispersion likely contribute to the overall differences in the gut microbiome between males and workers and queens and workers.

**Table 3:** Results from pairwise comparisons of dispersion from the centroid between honey bee castes (PERMDISP based on Bray-Curtis dissimilarity matrix of read counts).

	Males	Foragers	Nurses	Queens
**Males**		t = 9.26	t = 6.21	t = 1.43
		p = 0.0001	p = 0.0001	p = 0.4546
**Foragers**			t = 0.64	t = 3.74
			p = 0.6013	p = 0.0005
**Nurses**				t = 2.39
				p = 0.0451
**Queens**				

P-values are Monte-Carlo estimates from 9,999 unrestricted permutations of the raw data.

Shannon’s diversity index, which accounts for both abundance and evenness of OTUs in each sample, was significantly different between groups (ANOVA: F = 49.15, p<0.0001, n = 60; Fig 1C). Nurses and foragers had the highest diversity index, and both had significantly higher diversity than males or queens (post-test: p<0.05), but were not significantly different from each other.

We tested the strength of the association between the multivariate OTU data and caste with a canonical analysis of principal coordinates. There was a clear separation of castes by canonical axes 1 and 2, which together explain 39% of the variance (Fig 1D). Cross-validation, which uses discriminant analysis to classify samples based on multivariate data, indicated that OTU frequencies correctly predicted group membership for 47 of 60 (78.3%) individuals. Microbiome data was most predictive of male (93.8% correct), nurse (72.2% correct), or forager (77.3% correct) caste membership, but the strength of this association was lower for queens (50% correct). The five foragers that were misidentified were predicted to be nurses, and four of the five misidentified nurses were predicted to be foragers. These results emphasize the similarity between the two types of workers. One male was classified as a nurse, and two queens (one young, one old) were classified as males.

### Relative abundance of previously characterized phylotypes varies among castes

**Fig 2:**
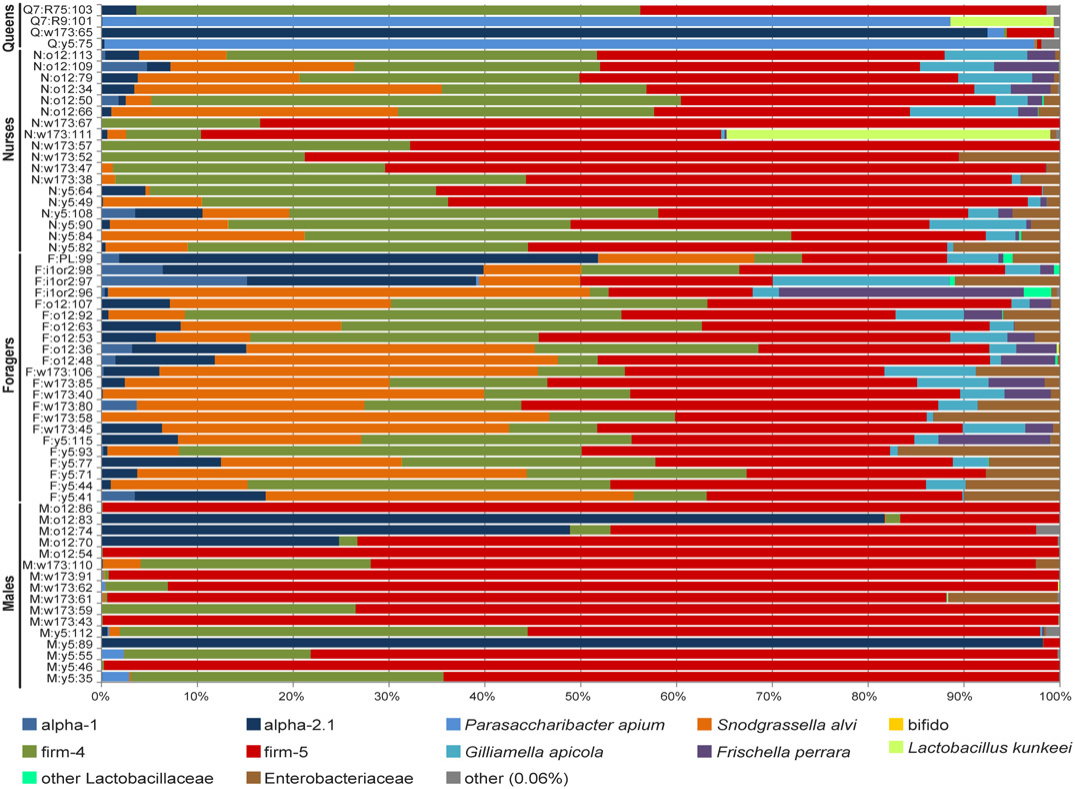
Family-level taxonomic composition of individual hindgut microbiomes in honey bees. Proportion of each taxa in total microbiome is represented by proportion of colored bar. Each bar represents one individual. Labels are as in Fig 1.

The microbiomes of nurses and foragers were highly similar to those observed in previous studies of worker honey bee microbiomes. Of the 4,402,282 sequences from nurses and foragers, 4,401,036 (99.97%) belonged to OTUs classified as phylotypes previously characterized in honey bees or were from bacterial families previously detected in honey bees (e.g., other Lactobacillaceae) (Fig 2).

**Fig 3:**
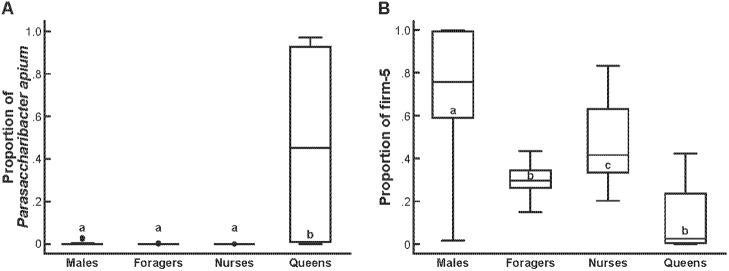
Caste differences in specific bacterial phylotypes. **(A) *P*. *apium*, (B) firm-5.** Statistical differences were tested with ANOVA, followed by post-tests for pairwise differences. Letters indicate significant pairwise differences (p<0.05).

The hindgut microbiome of males and queens have not been previously characterized. Our results show that males and queens have most of the same bacteria in their hindguts as workers (Fig 2). Over 99% and 98.87% of the total reads from males and queens, respectively, were classified as bacteria previously detected in honey bees. Despite this similarity in overall composition, the relative proportion of certain bacteria did vary significantly among castes. For example, queens had a higher relative abundance of the alpha-proteobacteria *Parasaccharibacter apium* than the other castes (ANOVA F = 17.89, p<0.0001, n = 60; post-tests p<0.05, Fig 3A). Caste membership was a significant predictor of *P*. *apium* abundance (LR χ^2^ = 10.51, n = 60, p = 0.001). Only three (7.5%) workers had more than ten sequences of *P*. *apium*; all three workers were from the indoor colonies. The large abundance of *P*. *apium* was not characteristic of all queens, however. One of the young 7-d old queens had no detectable *P*. *apium*, but the two queens that did have high *P*. *apium* (one old, one young) had more than 13 × higher abundance of *P*. *apium* than any other individual.

The *Lactobacillus* species firm-5 was the most abundant phylotype in males, foragers, and nurses, representing 75.7%, 30.4%, and 49.4% of total sequences from these castes, respectively. Firm-5 was the fourth most abundant bacteria found in queens, however, with *P*. *apium*, alpha-2.1, and firm-4 ranking ahead of firm-5 (Fig 2). Caste membership was a significant predictor of firm-5 abundance (LR χ^2^ = 6.97, n = 60, p = 0.0083). Males had significantly higher relative abundance of firm-5 than any other group (ANOVA F = 18.20, n = 60, p<0.0001, post-tests p<0.05, Fig 3B). Nurses had a significantly higher proportion of firm-5 than foragers (Fig 3B).

## Discussion

Work with primates has demonstrated that shared social communities and shared environments contribute to a common gut microbiome [29,44,45]. By contrast, the gut microbiome is highly variable within and across populations and species of the solitary fruit fly, *Drosophila* [46]. It has been previously hypothesized that sociality could facilitate host-symbiont fidelity of a shared microbiome among hosts [16–18]. In support of this hypothesis, microbiomes sampled from some social species of bees, such as honey bees and bumble bees, tend to be host-specific [17,21,47], but see [48] for exceptions in other social species. In addition, a remarkably consistent microbiome has been found among worker honey bees from different hives, states, countries, and subspecies [17,23–28].

Our results support the hypothesis that sociality facilitates the development of a shared microbiome. We identified of a core set of microbial taxa detected among at least one bee of each caste, consisting of previously characterized phylotypes found in honey bee workers. In addition, the microbiomes of honey bee workers in this study are similar to results of previous studies that also analyzed workers (e.g., compare nurses and foragers in Fig 2 to Moran et al. 2012 [24] Fig 1 and Sabree et al. 2012 [25] Fig 1).

The similarity between nurse and forager microbiomes is surprising, because variation in the microbiome is generally associated with host diet, behavior, and physiology [49,50], all of which vary between these two groups of specialized bees. Changes in diet and gut physiology accompany the age-related transition from hive work as nurses to foraging outside of the hive [11]. Shifts in bacterial abundance in honey bee workers have been observed to correspond to a peak in pollen consumption [47]. Nurses in our study had significantly more *Lactobacillus* species (firm-5) than foragers. This group of bacteria function in carbohydrate metabolism and transport [16]. Thus, differences in the relative abundance of these bacteria may reflect variation in the hindgut environment due to differences in the diets of nurses and foragers. Nine different OTUs were classified as firm-5, indicating that there is a great deal of strain diversity within this phylotype, as has been described for *Gilliamella apicola* and *Snodgrassella alvi* [51]. When the effect of these and other highly abundant OTUs was reduced, the microbiomes of nurses and foragers in our study were not significantly different from one another (Fig 1B, Table 2). This is consistent with previous sampling of honey bee gut microbiomes over their adult life span [20] and a recent sampling of forager whole gut microbiomes [52]. Similarly, activation of the immune system by parasites, dietary changes, and variation in body size (which is correlated with age) did not influence the microbiome of individual bumble bee (*Bombus terrestris*) workers [19]. These results suggest that the community composition of gut bacteria among workers in social bees is stable.

The stability of the worker microbiome may be due to frequent re-inoculation by interacting nestmates. Nurses exchange the most food with foragers, which could explain why they have such similar microbiomes, despite drastic differences in behavior, age, and physiology. Food is exchanged near the entrance to the hive, where nectar-laden returning foragers are unloaded by receiver bees and foragers are fed by nurses before each foraging flight [53]. For example, within four hours of giving six foragers labeled sugar water, 62% of the foragers and 20% of the total worker population received some of this food [54]. Another study showed that, of the total food produced and distributed by 100 nurses in one night, queens received 0.15%, drones 5.36%, but the majority (52.28%) went to other workers [55]. This relatively high exchange of food between nurses and foragers may reinforce microbiome similarities between the two groups.

This central role for nurses and foragers in filtering input from the external environment may also explain differences in hindgut microbial communities among castes. We found large differences in hindgut microbiomes between males and workers (Fig 2, Table 1) and marginal differences between queens and workers, when the influence of the most abundant OTUs was reduced (Table 2). Worker hindguts also have significantly higher microbial diversity than queens and males (Fig 1C). We suggest that this may reflect a central role for these individuals in the maintenance of the colony microbiome. Increased strain diversity may be the result of regular exchange of environmentally derived sources between returning foragers and receiving nurses. Foragers are likely responsible for introducing new strains of bacteria from the environment to the colony, which may then be filtered and distributed among colony members by nurses. Worker bees lack a gut microbiome when they first eclose, and will not develop a microbiome if they are kept isolated from the colony [56]. Nurses feed all colony members with food made in specialized glands in the head (hypopharyngeal glands), and thus the entire colony’s nutrients, and possibly microbes, pass through nurses. For example, we did not detect *P*. *apium* in the hindguts of nurses, but it was highly abundant in queens. Previous research has found this bacterium in the crops of foragers [52] and the crops and hypopharyngeal glands of nurses, as well as in the royal jelly they feed to the queens [40]. Other bacteria (e.g., *S*. *alvi*, *G*. *apicola*, and *F*. *perrara*) may only be transferred through direct contact with nurses or their fecal material [56]. All individuals in a colony likely receive a diverse suite of bacteria from nurses, but most bacteria either do not persist or are extremely rare in all colony members.

Some caste-related differences in microbiome composition may be related to differences in reproductive activity. *P*. *apium* was significantly more abundant in queens than all other castes, and was nearly absent from most workers, who do not reproduce (Fig 3A). Genes expressed in strains of these bacteria found in workers are linked to ferrodoxins and oxidoreductases associated with metabolism as well as membrane/envelope biogenesis [16,57], which are important components of oogenesis. This phylotype has also been detected in solitary bee species [17], the captured females of which would also likely be reproductive. These bacteria may, therefore, be useful for converting large amounts of dietary protein to egg production.

Our measures of diversity are necessarily relative, as we did not measure absolute bacterial numbers in each sample. Nonetheless, our results reveal significant differences in the relative proportion of bacteria. For example, the phylotype most commonly found in workers is reduced, relative to alpha-proteobacteria in queens. Our findings are consistent with accumulating evidence of the role of microbes mediating host behavior and host behavior influencing the microbial symbiont community [2,58,59].

Chemical communication within the beehive may be particularly affected by filtering symbiotic microbes through workers [2,60]. *Lactobacillus plantarum* influences mate preferences in *Drosophila melanogaster*, possibly through alterations to cuticular hydrocarbon sex pheromones [7]. Pheromones also play a large role in honey bee social behavior. By distributing a core microbiome throughout the colony, nurses may facilitate nestmate recognition. Testing this and other potential functions of the microbiome in host social behavior requires further study. Our study provides evidence that host-symbiont dynamics may be an additional level of biological organization at which social behavior is regulated.

## Supporting Information

S1 Appendix Representative sequences of each OUT. (TXT)

S1 Fig Histograms showing the distribution of the number of reads across samples. Bins are of width 10,000 reads. In general, the nurse samples have the largest number of reads, while the queen samples have the least. Males—green bars, Foragers—purple bars, Nurses—teal bars, 7d old queens—pink bars, Queens—orange bars.
(TIFF)

S2 Fig Non-metric multi-dimensional scaling plot of OTU frequency, based on a Bray-Curtis dissimilarity matrix. Stress value: 0.17. Symbols are as in Fig 1.
(TIFF)

S1 Table Individual bee data and sequence identifiers. (PDF)

S2 Table Results of semi-parametric analysis of variance. (PDF)
