# Supplementary figures and images for: Caste-Specific Differences in Hindgut Microbial Communities of Honey Bees (Apis mellifera)
Source: PLoS One. 2015 Apr 15;10(4):e0123911. doi: 10.1371/journal.pone.0123911 (PMC4398325; doi:10.1371/journal.pone.0123911)

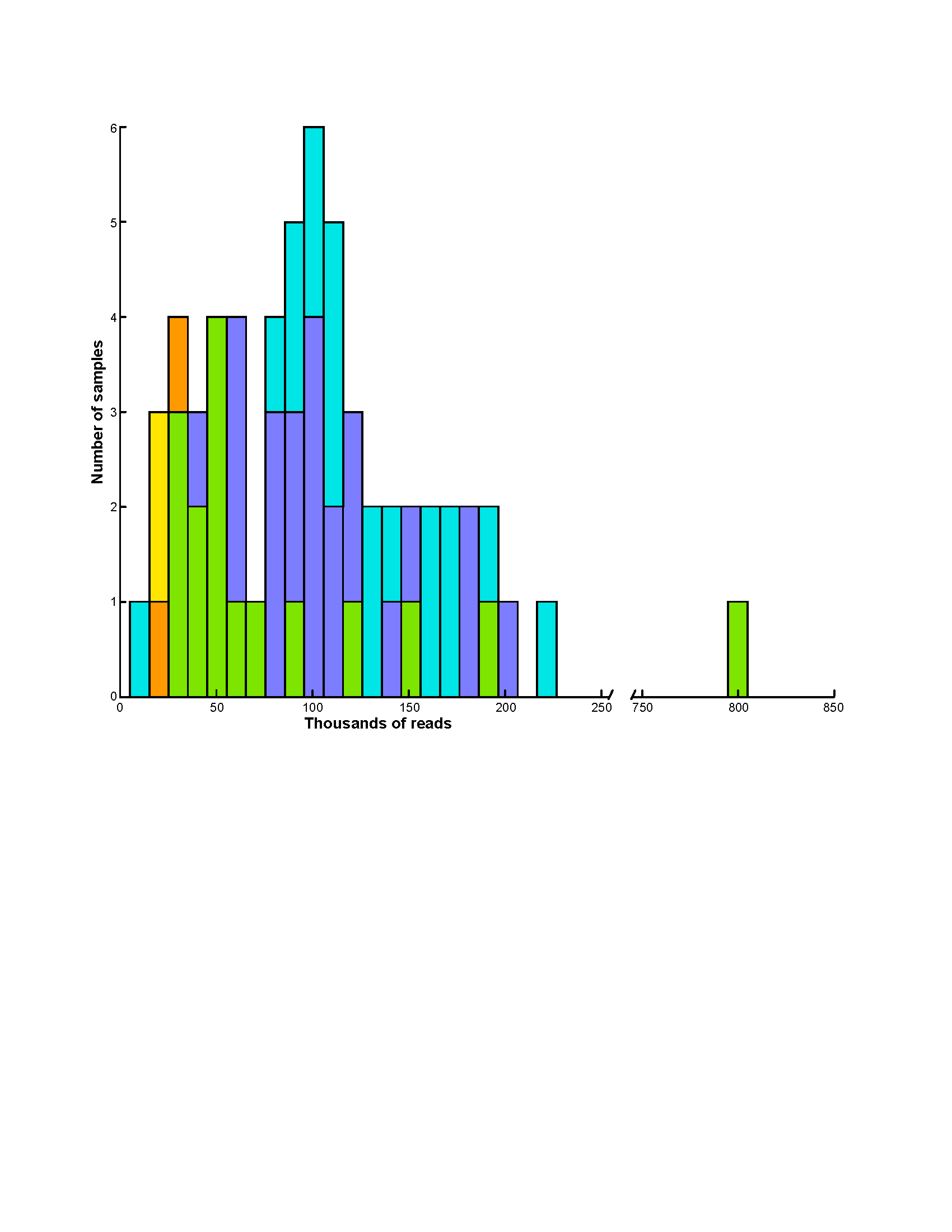

Supplement: S1 Fig — Bins are of width 10,000 reads. In general, the nurse samples have the largest number of reads, while the queen samples have the least. Males—green bars, Foragers—purple bars, Nurses—teal bars, 7d old queens—pink bars, Queens—orange bars. (TIFF) [file pone.0123911.s002.tiff]

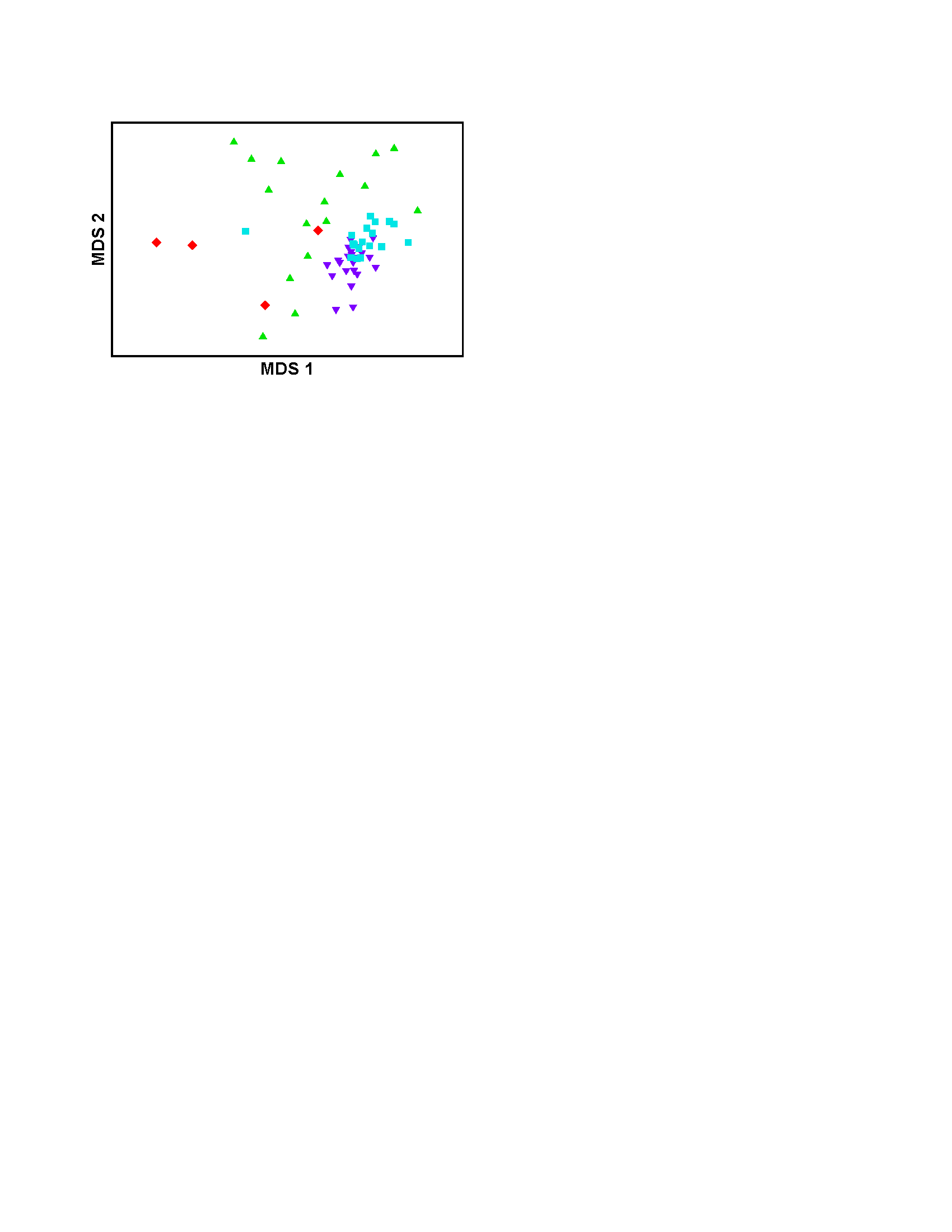

Supplement: S2 Fig — Stress value: 0.17. Symbols are as in Fig 1. (TIFF) [file pone.0123911.s003.tiff]
