# Supplementary material for: Caste-Specific Differences in Hindgut Microbial Communities of Honey Bees (Apis mellifera)
Source: PLoS One. 2015 Apr 15;10(4):e0123911. doi: 10.1371/journal.pone.0123911 (PMC4398325; doi:10.1371/journal.pone.0123911)
Supplement: S1 Table — (PDF) [file pone.0123911.s004.pdf]

Caste-specific differences in hindgut microbial communities of honey bees (*Apis mellifera*)

Karen M. Kapheim, Vikyath D. Rao, Carl J. Yeoman, Brenda A. Wilson, Bryan A. White, Nigel Goldenfeld, Gene E. Robinson

Supplementary Table S1. Individual bee data and sequence identifiers

Sample ID	Caste	Colony	Accession
F:i1or2:96	Forager	indoor colony 1 or 2	SRS800921
F:i1or2:97	Forager	indoor colony 1 or 2	SRS721657
F:i1or2:98	Forager	indoor colony 1 or 2	SRS800976
F:PL:99	Forager	pooled samples from indoor colony 1 and 2	SRS800977
F:o12:107	Forager	o12	SRS800970
F:o12:36	Forager	o12	SRS800934
F:o12:48	Forager	o12	SRS800940
F:o12:53	Forager	o12	SRS800974
F:o12:63	Forager	o12	SRS800968
F:o12:92	Forager	o12	SRS800945
F:w173:106	Forager	w173	SRS800936
F:w173:40	Forager	w173	SRS800938
F:w173:45	Forager	w173	SRS800942
F:w173:58	Forager	w173	SRS800932
F:w173:80	Forager	w173	SRS800941
F:w173:85	Forager	w173	SRS800943
F:y5:115	Forager	y5	SRS800944
F:y5:41	Forager	y5	SRS800933
F:y5:44	Forager	y5	SRS800971
F:y5:71	Forager	y5	SRS800935
F:y5:77	Forager	y5	SRS800967
F:y5:93	Forager	y5	SRS800930
M:o12:54	Male	o12	SRS800892
M:o12:70	Male	o12	SRS800919
M:o12:74	Male	o12	SRS800920
M:o12:83	Male	o12	SRS800888
M:o12:86	Male	o12	SRS800890
M:w173:110	Male	w173	SRS800893
M:w173:43	Male	w173	SRS800886
M:w173:59	Male	w173	SRS800885
M:w173:61	Male	w173	SRS721656
M:w173:62	Male	w173	SRS800895
M:w173:91	Male	w173	SRS800897
M:y5:112	Male	y5	SRS800917
M:y5:35	Male	y5	SRS800916
M:y5:46	Male	y5	SRS800891
M:y5:55	Male	y5	SRS721655
M:y5:89	Male	y5	SRS800887
N:o12:109	Nurse	o12	SRS800948
N:o12:113	Nurse	o12	SRS800975
N:o12:34	Nurse	o12	SRS800939
N:o12:50	Nurse	o12	SRS800928
N:o12:66	Nurse	o12	SRS800937
N:o12:79	Nurse	o12	SRS800947
N:w173:111	Nurse	w173	SRS721653
N:w173:38	Nurse	w173	SRS800929
N:w173:47	Nurse	w173	SRS800924
N:w173:52	Nurse	w173	SRS800922
N:w173:57	Nurse	w173	SRS800925
N:w173:67	Nurse	w173	SRS800927
N:y5:108	Nurse	y5	SRS800973
N:y5:49	Nurse	y5	SRS800923
N:y5:64	Nurse	y5	SRS800926
N:y5:82	Nurse	y5	SRS800946
N:y5:84	Nurse	y5	SRS800931
N:y5:90	Nurse	y5	SRS800972
Q:w173:65	Queen	w173	SRS800889
Q:y5:75	Queen	y5	SRS721654
Q7:R75:103	7 day old queen	R75	SRS800918
Q7:R9:101	7 day old queen	R9	SRS800884
