# Supplementary material for: Caste-Specific Differences in Hindgut Microbial Communities of Honey Bees (Apis mellifera)
Source: PLoS One. 2015 Apr 15;10(4):e0123911. doi: 10.1371/journal.pone.0123911 (PMC4398325; doi:10.1371/journal.pone.0123911)
Supplement: S2 Table — (PDF) [file pone.0123911.s005.pdf]

Caste-specific differences in hindgut microbial communities of honey bees (*Apis mellifera*)
Karen M. Kapheim, Vikyath D. Rao, Carl J. Yeoman, Brenda A. Wilson, Bryan A. White, Nigel Goldenfeld, Gene E. Robinson

Supplementary Table S2. Results of semi-parametric analysis of variance

	Df	SS	MS	Pseudo-F	p-value
Caste	3	40618	13539	4.1459	0.0001
Colony	5	19800	3959.9	2.2204	0.0001
Caste x Colony	5	17379	3475.7	1.9489	0.0007
Residual	46	82038	1783.4		
Total	59	1.6449E5			
